# Gun Violence Exposure and the Perceived Need to Carry a Firearm: The Role of Community Violence Intervention Awareness

**DOI:** 10.1007/s11524-026-01115-w

**Published:** 2026-07-31

**Authors:** Stephen N. Oliphant, Shaun Bhatia, Jessica Bishai, Jennifer K. Paruk, Daniel B. Lee, Daniel C. Semenza, Andrew V. Papachristos, Justin E. Heinze

**Affiliations:** 1 Institute for Firearm Injury Prevention, University of Michigan, 1109 Geddes Ave, Ann Arbor, MI 48109 USA; 2 Institute for Policy Research, Northwestern University, 2040 Sheridan Road, Evanston, IL 60208 USA; 3 School of Public Health, Health Behavior and Health Equity, University of Michigan, 1415 Washington Heights, Ann Arbor, MI 48109 USA; 4 New Jersey Gun Violence Research Center, Rutgers University, 683 Hoes Ln W, Piscataway, NJ 08854 USA; 5 School of Social Work, University of Michigan, 1080 S University Ave, Ann Arbor, MI 48109 USA; 6 Department of Sociology, Anthropology, and Criminal Justice, Rutgers University, 405-7 Cooper Street, Camden, NJ 08102 USA; 7 Department of Urban-Global Public Health, Rutgers University, 683 Hoes Ln W, Piscataway, NJ 08854 USA; 8 Department of Sociology, Northwestern University, 1810 Chicago Ave, Evanston, IL 60208 USA

**Keywords:** Gun violence, Violence exposure, Community violence intervention, Firearm carrying, Mental health, Safety perceptions

## Abstract

Gun violence exposure (GVE) is associated with adverse mental health outcomes and firearm carrying. The extent to which community-led programs aimed at reducing violence counteract these effects is unknown. This study sought to examine (1) the relationships between GVE, anticipated risk of assault, psychological distress, and the perceived need to carry a firearm for protection and (2) whether awareness of community violence intervention (CVI) efforts in one’s neighborhood moderates these associations. We used weighted survey data from a representative sample of 2,285 Detroit residents collected in 2024. Structural equation modeling approaches were used to estimate relationships. We found that past 6-month GVE was positively associated with psychological distress, anticipated assault risk, and a greater perceived need to carry a firearm for protection. GVE was also indirectly associated with a greater perceived need to carry a firearm through increased anticipation of assault. Path coefficients from GVE to psychological distress, anticipated assault risk, and the perceived need to carry a firearm were smaller among the CVI awareness group, and, apart from the association between GVE and anticipated assault risk, not significant. The positive association between GVE and psychological distress was significantly weaker among those who reported being aware of CVI in their neighborhood. Overall, these findings suggest that CVI awareness may be associated with a weaker relationship between GVE and mental well-being and safety-related perceptions. Additional research that examines broader health and safety-related outcomes is needed to more fully characterize the potential benefits of CVI, including among the broader community.

**Supplementary Information:**

The online version contains supplementary material available at https://doi.org/10.1007/s11524-026-01115-w.

## Background

Firearm violence imposes a substantial toll on victims, their families, and the broader community [1, 2]. A growing body of research highlights the harmful effects of gun violence exposure (GVE), including both direct (e.g., being shot at or witnessing gun-related harms) and indirect exposure (e.g., knowing someone who was shot at) [3]. At the individual level, GVE is theorized to contribute to negative physiological processes, such as chronic stress, increased anxiety, and changes to threat perceptions. In turn, these responses shape mental and physical health by hindering healthy behaviors or facilitating harmful coping responses, such as substance use [3]. Studies have found that survivors of shootings experience worse mental health, as evidenced by new mental health diagnoses [4] and poorer mental health-related quality of life [5]. Adverse effects extend to those exposed to gun violence but not injured, as witnessing or hearing about a neighborhood shooting has been associated with poor self-reported mental health among Black adults [6] and quality of life among adults across nine states [7]. In addition to cumulative exposure, both the frequency and recency of GVE are associated with worse mental health outcomes, including posttraumatic symptomatology, depression, and suicidal ideation [8, 9].

Similar associations are presented in studies that assessed vicarious or indirect exposure, where a friend or family member being shot in the past year and knowing someone who was fatally shot were linked to poorer mental health, including psychological distress, mental health diagnoses, and suicidal ideation [4, 9–11]. Other findings that hearing gunshots in the past year or two were associated with increased mental health service utilization and suicidal ideation, respectively, suggest that the adverse effects of less direct exposures may persist over time [9].

Evidence from quasi-experimental studies that leverage temporal and geographic variation in shootings and homicides also highlights the negative impact of indirect exposure to violence [12]. Among children aged 0–19 years, residential proximity to shootings in Philadelphia was associated with increased odds of presenting to the emergency department for mental health-related concerns in the following weeks and months [13]. Likewise, geographic proximity to violent crime incidents, mass shootings, and homicides has been associated with increased mental health problems [14], poorer academic performance [15], and lower performance on cognitive assessments [16], respectively.

In addition to the negative outcomes described above, GVE is also associated with firearm carrying [17–20]. The perceived need for protection, a primary reason people carry firearms [19, 21, 22], may partially explain this relationship. Although carriage is itself a risk factor for gun assault victimization [23, 24], those directly or indirectly exposed to gun violence might carry firearms as a defensive means to prevent future victimization. This type of response to GVE or anticipated assault is consistent with the theorized coping behaviors following experienced, vicarious, or anticipated strain within general strain theory [25].

Despite the established associations of GVE with psychological distress and firearm carrying, potential mediating factors for these relationships are not well understood. First, qualitative data suggest that anxiety can stem from a fear of violence [26], which may suggest an indirect association between GVE and psychological distress vis-à-vis anticipated assault. While anticipatory violence has been associated with worse self-reported physical health, the evidence of a connection specifically to mental health is mixed [6, 27]. Second, psychological distress may partially mediate the relationship between GVE and firearm carrying perceptions. Among male youth convicted of serious offenses, those with high psychological distress and violence exposure were the most likely to carry guns in the future [28]. Third, GVE may increase anticipation of negative outcomes (e.g., assault victimization), in turn influencing perceptions around needing to carry firearms for protection. Although focused on educational expectations, Lee et al. [29] found that negative future expectations mediated the longitudinal association between violence exposure and adolescent firearm carrying. Given that carriage among those who have been exposed to violence is linked to an increased risk of future firearm violence [30], interventions that counteract the psychological and behavioral effects of GVE may be effective in reducing firearm-related harm within communities.

Community violence intervention (CVI) is a promising approach to reduce firearm violence and improve public safety. While specific details of CVI vary, the approach generally seeks to prevent violence by mediating conflicts, connecting individuals at risk of violence to unmet social needs, offering mentorship, and promoting nonviolence. Motivating this approach is the notion that those embedded in communities impacted by violence are credible messengers who can help disrupt retaliatory violence and change norms around firearm use. As described by Hureau and Papachristos [31], these “outreach workers” are “active builders of social capital, bridgers of social and institutional networks, and supporters of neighborhood mechanisms of informal social control and collective efficacy” (p. 433).

Hureau and Papachristos [31] argue that research on outreach work has prioritized narrow evaluations centered around program effectiveness at reducing various violence metrics. The resulting evidence has been mixed. An evaluation of Safe Streets in Baltimore by Buggs et al. [32] found no evidence of sustained reductions in homicides and non-fatal shootings at the neighborhood level. Ross et al. [33] found that participation in an outreach program in Chicago was not associated with reduced firearm victimization risk; however, those who completed programming were 73% less likely to be arrested for a violent crime. These findings suggest that CVI may have greater influence on individual-level behaviors—and perceptions theorized to shape behavior—than on victimization risks posed by factors beyond an individual’s control [33]. In addition, while evaluations focused on shooting incidence, arrests, and related metrics are informative, there is a need to examine a broader set of outcomes relevant to public safety and well-being [31, 34]. Outcomes that theoretically facilitate violence reduction, such as changes in perceptions and behaviors, are appropriate starting points [31]. Perceptions around firearm carrying are particularly relevant given that the conceptual framework of CVI approaches like Cure Violence relies in part on changing norms around gun use [35]. Research investigating these types of intermediate outcomes could begin to uncover important public health and safety benefits of CVI that are not addressed in existing evaluations. Moreover, research that considers potential impacts among the broader community could reveal additional insights beyond those uncovered in conventional evaluations of CVI participants. To date, no studies have analyzed whether awareness of CVI efforts moderates the associations between GVE and adverse outcomes.

Awareness of neighborhood CVI efforts may buffer against the adverse consequences of GVE in two ways. First, knowing that community-led efforts to reduce violence are taking place may positively affect one’s mental well-being and sense of community. Changes to the physical environment in the form of vacant land remediation have been linked to improvements in mental health [36], collective efficacy [37], and perceived safety [38]; likewise, interventions that are aimed at improving community social processes (e.g., informal social control, social capital, and collective efficacy) may also generate psychological benefits. Second, knowledge of CVI efforts may alter safety-related perceptions, including one’s own perceived risk of victimization and the extent to which preemptive measures, such as firearm carrying, are necessary. These perceptions may be less pronounced for those who have been exposed to gun violence but are aware of ongoing CVI efforts in their community.

In Detroit, specifically, CVI is characterized by conflict mediation, wraparound services to connect at-risk individuals to social services, and community outreach to change social norms around violence [39]. Prior to June 2025, when coverage areas expanded, the six CVI organization “zones” covered 22 square miles, or approximately 16% of the city [40]. However, 2024–2025 data demonstrate that the 12,113 total service events provided by CVI organizations—comprising 22,522 total services—extended well beyond these zones [39].

To better understand the complex relationships between GVE, mental health, and safety-related perceptions, and the potential role that awareness of CVI efforts in Detroit may play in moderating these associations, we tested the following hypotheses:

- Hypothesis 1: GVE is positively associated with psychological distress, anticipatory assault risk, and the perceived need to carry a firearm for protection.
- Hypothesis 2: Anticipatory assault risk and psychological distress mediate the association between GVE and the perceived need to carry a firearm for protection.
- Hypothesis 3: Awareness of neighborhood CVI efforts attenuates the adverse associations between GVE and psychological distress and safety-related perceptions.

## Methods

### Data

We obtained deidentified Detroit Metro Areas Community Study (DMACS) Wave 20 data from Deep Blue Data [41], a publicly available data repository. Since 2016, DMACS surveys have assessed Detroiters’ perceptions on many issues, including neighborhoods, safety, policing, city investment, housing, and transportation, as well as survey-specific topics such as COVID-19 and elections [42].

The Wave 20 survey collected information from a representative panel of Detroit residents in June–August 2024. Invitations were sent to 3012 previously enrolled respondents and 4000 randomly selected households, resulting in a study sample of 2450 respondents [41]. Males with less than a college education were offered a $40 incentive whereas all other participants were offered $25. The larger incentive structure was intended to improve participation among this low-response group [41]. Our analytic sample included 2285 respondents with complete data on the variables incorporated in our model (93.3% of survey respondents). The DMACS team used a two-stage process involving post-stratification and raking to calibrate weights to match estimated distributions of Detroit population demographics (age, gender, race, education, income) from the 2022 American Community Survey. Additional information regarding survey procedures can be found at the study’s website [42] and from study materials [41].

### Measures

Our outcome variable, perceived need to carry a firearm for protection in one’s neighborhood, was assessed using a single item. Respondents indicated their level of agreement with the statement: “In this neighborhood, it is sometimes necessary for people to carry a gun to protect themselves or their family.” The Likert response scale ranged from strongly disagree to strongly agree (0–4), with higher values indicating a greater perceived need to carry.

Past six-month GVE was measured using two items that capture community-level and direct or secondary exposure [3, 20]. Respondents were asked (a) whether they had heard gunshots while in their neighborhood in the past six months (yes/no) and (b) whether they or anyone else had been shot at while in the neighborhood in the past six months. Respondents who reported not hearing gunshots and answered “no, I am not aware of this [someone being shot at] happening in my neighborhood” were coded as 0. Those who reported hearing gunshots in the past six months but did not report that they or someone else were shot at in their neighborhood (i.e., no direct or vicarious exposure) were coded as 1. Respondents who reported that they or someone else were shot at in their neighborhood were coded as 2. A higher value for GVE indicates more direct exposure to gun violence.

Anticipated assault risk was measured using four items, two of which have been used to assess anticipatory community violence [6]. Respondents answered on a five-point Likert scale how likely it is that they would be assaulted in the following ways in the next six months: (a) mugged; (b) shot at; (c) violently attacked; and (d) have their home broken into in a burglary. Higher values correspond to greater anticipated assault. The combined measure of anticipated assault risk exhibited excellent internal consistency (*α* = 0.90).

Psychological distress was assessed using two questions adapted from the GAD-7 anxiety scale [43]. Respondents indicated how often in the past seven days they had (a) felt nervous, anxious, or on edge and (b) not been able to stop or control worrying. Responses included less than 1 day, 1–2 days, 3–4 days, and 5–7 days. The mean value across the two items was used to capture psychological distress frequency. The combined item exhibited good internal consistency (*α* = 0.81).

Our moderating or group variable, awareness of CVI in one’s neighborhood, was derived from two conditional questions. First, respondents reported whether they were aware of any community-led programs or community organizations working to reduce violence in their neighborhood. Those who answered “yes” were asked, “Of these community-led programs working to reduce violence in your neighborhood, are any using mediation, mentoring, and/or de-escalation?” (consistent with the City of Detroit’s description of CVI strategies) [39]. Individuals who answered “yes” to both questions were coded as 1 for CVI awareness, whereas those who answered “no” to either question were coded as 0.

We included three sociodemographic variables as controls. Educational attainment included: (0) less than high school, (1) high school diploma or GED, (2) some college or associate’s degree, and (3) bachelor’s or graduate degree. Variables capturing race (0 = non-Black, 1 = Black) and ethnicity (0 = non-Hispanic, 1 = Hispanic) were also included. Household income and gender were excluded because they were not significantly associated with the perceived need to carry a firearm. We also excluded age because it resulted in markedly worse model fit. A sensitivity analysis including age as a control variable is included in the Supplemental Material.

### Analysis

We used structural equation modeling (SEM) to estimate the relationships between GVE, psychological distress, anticipated assault risk, and the perceived need to carry a firearm in Hypotheses 1 and 2. To test Hypothesis 3, we conducted a multigroup SEM to assess whether CVI awareness moderated the relationships tested in the first two hypotheses, with Group 1 comprising those who were not aware of CVI in their neighborhood (*n* = 2015) and Group 2 comprising those who were aware of CVI (*n* = 254). We also conducted a sensitivity analysis given that many respondents reported awareness of community-led programs or organizations working to reduce violence but were unaware if those efforts involved CVI. Those who indicated awareness of violence reduction programs but answered “no” or “don’t know” to the second question assessing CVI-specific awareness were introduced as a third group, resulting in the following categorizations: no awareness of community-led violence reduction programs or organizations (Group A, *n* = 1541; 67.9%), awareness of nonspecific violence reduction programs or organizations (Group B, *n* = 474; 20.9%), and awareness of CVI-specific violence reduction programs or organizations (Group C, *n* = 254; 11.2%). We used Wald tests to determine whether the direct, indirect, and total effects in the multigroup analyses were significantly different across groups. Specifically, we used the *testnl* Stata command to test inter-group equality of (1) direct path coefficients, (2) products of coefficients (i.e., indirect paths), and (3) the sum of the direct and indirect paths (i.e., total).

To account for the differential selection probability and to approximate Detroit’s population on demographic characteristics [41], we incorporated weights using the *svy* suite of commands for analyzing complex survey data. We used the standardized root mean square residual (SRMR) to assess goodness of fit for each model. We also examined the root mean square error of approximation (RMSEA), comparative fit index (CFI), and Tucker–Lewis index (TLI) from unweighted but otherwise identically specified models as additional assessments. Analyses were conducted using the maximum likelihood estimation method and the *sem* command in Stata 18 (StataCorp LP, College Station, TX).

## Results

Table 1 presents descriptive statistics from the weighted sample. Consistent with citywide demographics [44], 78.6% of respondents were Black and 6.7% were Hispanic. Nearly half (47.4%) somewhat or strongly agreed that it is sometimes necessary to carry a firearm for protection. Similarly, 49.9% reported hearing gunshots but no direct or vicarious shooting victimization in their neighborhood, while 22.1% reported that they or someone else had been shot at in their neighborhood. On average, Detroiters reported experiencing psychological distress less than 1–2 days per week and expected that it was somewhat unlikely that they would be assaulted in the next six months. Approximately 11% indicated awareness of CVI in their neighborhood. Gun violence exposure was similar among those reporting awareness of CVI in their neighborhood (47.8% heard gunshots only; 25.2% reported being shot at or knowing someone who was) and those unaware of local CVI efforts (50.1% and 21.9%, respectively).

**Table 1:** Weighted descriptive statistics of the analytic sample (*n* = 2,285)

Variable	%	Mean (SD)
**Perceived need to carry** (0–4)		2.30 (0.04)
0—strongly disagree	14.0	
1—somewhat disagree	10.8	
2—neither agree nor disagree	27.8	
3—somewhat agree	25.7	
4—strongly agree	21.7	
**Gun violence exposure** (0–2)		0.94 (0.02)
0—no exposure	28.1	
1—heard gunshots only	49.9	
2—direct/vicarious targeting	22.1	
**CVI awareness**	10.9	
**Psychological distress** (0–3)		0.76 (0.03)
**Anticipated assault risk** (0–4)		1.03 (0.03)
**Education** (0–3)		1.53 (0.03)
0—less than high school	15.3	
1—high school diploma or GED	34.1	
2—some college or associate’s	32.8	
3—bachelor’s or graduate degree	17.8	
**Black**	78.6	
**Hispanic**	6.7	
**Woman**	56.0	
**Age** (18–84+)		47.4 (0.52)
18–34 years	31.0	
35–54	32.3	
55–64	15.9	
65 +	20.8	
**Household income**		
< $10,000	14.4	
$10,000—$29,999	27.9	
$30,000—$49,999	20.5	
$50,000—$99,999	24.7	
$100,000 +	12.5	

### Overall SEM

Results from the overall SEM model are presented in the first column of Table 2. GVE was directly associated with greater anticipated assault risk (0.44, *p* < 0.001), greater psychological distress (0.20, *p* < 0.001), and a greater perceived need to carry a firearm (0.40, *p* < 0.001). For example, each one-unit increase in GVE was associated with a 0.40-unit increase in the perceived need to carry a firearm on the 0–4 scale described above. GVE was also indirectly associated with psychological distress (0.06, *p* < 0.001) and a perceived need to carry a firearm (0.10, *p* < 0.001). Anticipated assault risk was associated with psychological distress (0.14, *p* < 0.001) and perceived need to carry a firearm (0.18, *p* < 0.001), whereas the relationship between psychological distress and perceived need to carry was only marginally significant (0.07, *p* = 0.10). Of the three indirect paths between GVE and perceived need to carry, only the single-mediator path (GVE → anticipated assault risk → perceived need to carry a firearm) was significant (0.08, *p* < 0.001).

**Table 2:** Direct and indirect path estimates in the overall and multigroup models

	Overall		Group 1:No CVI Awareness		Group 2: CVI Awareness	
	*b*	*p*	*b*	*p*	*b*	*p*
**Psychological distress**						
GVE → Psychological distress	0.20	<.001	0.22	<.001	0.04	.64
Anticipated assault → Psych. distress	0.14	<.001	0.15	<.001	0.04	.49
GVE → Anticipated assault → Psych. distress	0.06	<.001	0.07	<.001	0.02	.50
**Anticipated assault risk**						
GVE → Anticipated assault risk	0.44	<.001	0.45	<.001	0.38	<.001
**Perceived need to carry**						
GVE → Carry	0.40	<.001	0.43	<.001	0.27	.13
Psychological distress → Carry	0.07	0.10	0.08	0.12	0.03	.81
Anticipated assault risk → Carry	0.18	<.001	0.18	<.001	0.05	.66
GVE → Psych. distress → Carry	0.01	.12	0.02	.14	0.00	.83
GVE → Anticipated assault → Carry	0.08	<.001	0.08	.001	0.02	.66
GVE → Anticipated assault → Psych. distress → Carry	0.00	.12	0.01	.14	0.00	.81
SRMR	0.035		0.030			

Goodness of fit statistics for unweighted but identically specified models are as follows: Overall – RMSEA = 0.063, CFI = 0.934, TLI = 0.836; Multigroup – RMSEA = 0.062, CFI = 0.938, TLI = 0.845

### Multigroup Moderation

**Fig. 1:**
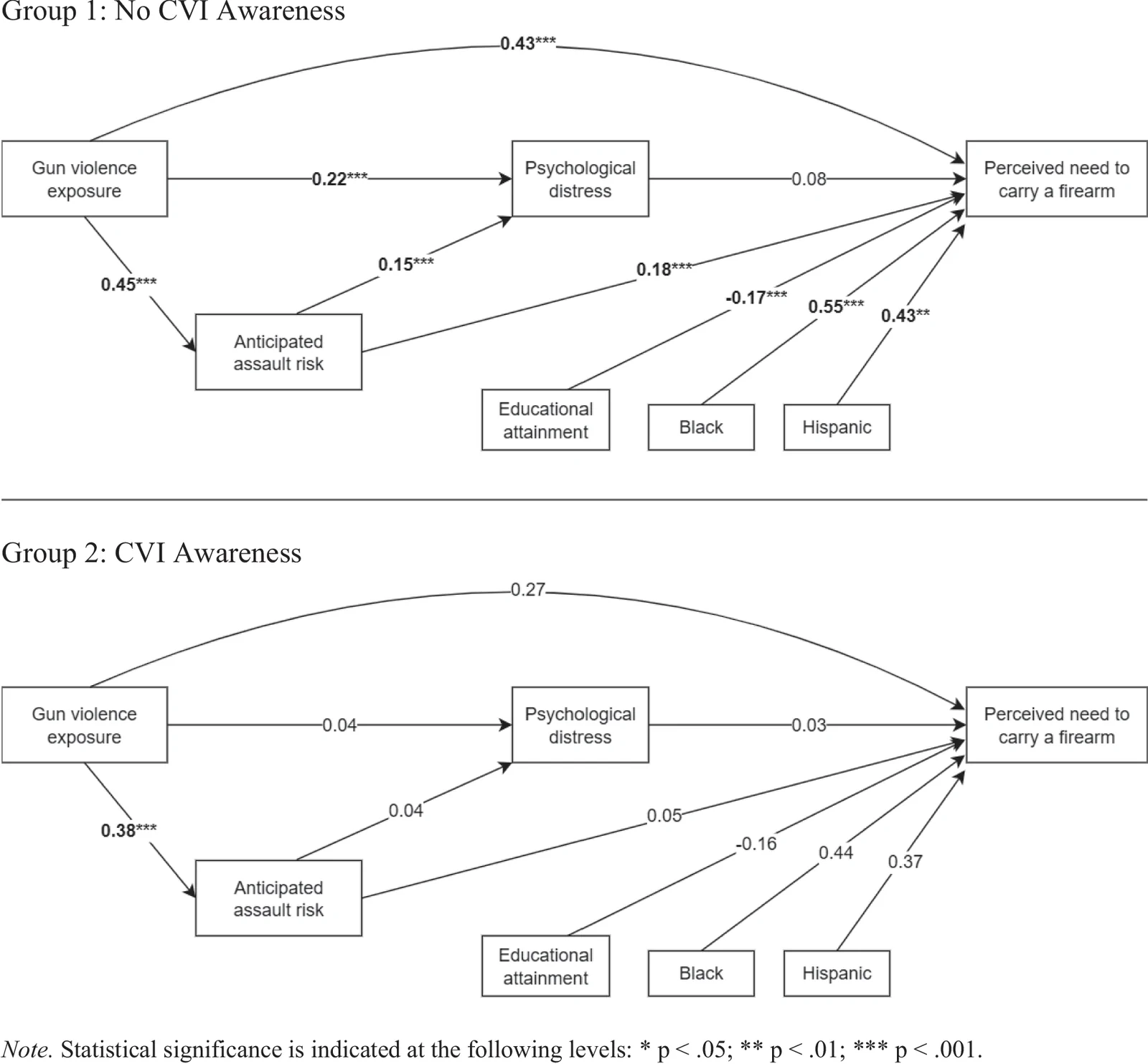
Multigroup structural equation model of the perceived need to carry a firearm among those who did not report awareness of CVI in their neighborhood (Group 1) and those who reported CVI awareness (Group 2)

Results from the multigroup SEM comparing pathways by CVI awareness are illustrated in Fig. 1 and presented in Table 2. Among those who did not report being aware of CVI in their neighborhood, GVE was positively associated with anticipated assault risk (0.45, *p* < 0.001), psychological distress (0.22, *p* < 0.001), and a perceived need to carry a firearm (0.43, *p* < 0.001). GVE was also indirectly associated with a perceived need to carry a firearm through anticipated assault risk among this group (0.08, *p* < 0.001). Conversely, all path coefficients were smaller among the CVI group, and apart from the association between GVE and anticipated assault risk (0.38, *p* < 0.001), not statistically significant.

Results from the Wald tests to assess significant differences between Groups 1 and 2 are presented in Table 3. The relationship between GVE and psychological distress differed significantly across groups, such that the association was weaker among those who were aware of CVI in their neighborhood (*p* = 0.045). Neither the direct (*p* = 0.392) nor indirect (*p* = 0.132) path estimates from GVE to perceived need to carry indicated significant group-level differences.

**Table 3:** Adjusted Wald tests of multigroup path estimates

Path	*p*
**Direct**	
**GVE → psychological distress**	**0.045**
Assault risk → psychological distress	0.109
GVE → assault risk	0.505
GVE → carry	0.392
Psychological distress → carry	0.726
Assault risk → carry	0.304
**Indirect**	
GVE → assault risk → psychological distress	0.060
GVE → psychological distress → carry	0.204
GVE → assault risk → carry	0.221
GVE → assault risk → psych. distress → carry	0.237
Assault risk → psych. distress → carry	0.253
GVE → psychological distress → carry	0.132
GVE → assault risk → carry	
GVE → assault risk → psych. distress → carry	
**Total**	
GVE → carry (direct and indirect paths)	0.162

### Sensitivity Analyses

In a sensitivity analysis, significant direct and indirect path estimates between GVE and the perceived need to carry were limited to those who were not aware of any violence reduction programs (Group A) and those who were aware of nonspecific violence reduction programs (Group B) (Table A1). In contrast, the path estimates among those who were aware of CVI (Group C) were smaller and not statistically significant. Wald test results indicated that there was a significant difference in the direct association of GVE with the perceived need to carry a firearm between Groups B and C (*p* = 0.006), such that the positive association was smaller among those who were aware of CVI-specific programs.

Results from sensitivity tests involving minor changes to model specification are presented in the Supplemental Material (Figures A1, A2). The inclusion of additional pathways from educational attainment to the mediating variables resulted in negligible changes to path coefficients (Figure A1). Likewise, including age as a control variable did not substantively change the path coefficients for either group despite worsening model fit (Figure A2). Finally, a comparison of maximum likelihood and asymptotic distribution-free estimation using unweighted data produced nearly identical path estimates and standard errors (Table A2).

## Discussion

Consistent with the patterns described in the GVE framework proposed by Semenza and Kravitz-Wirtz [3], we found that more direct GVE in the past six months was associated with more frequent psychological distress in the past week, as well as greater anticipation of assault victimization in the next six months and a greater perceived need to carry a firearm for protection. GVE was also indirectly associated with a greater perceived need to carry a firearm through increased anticipation of assault. Notably, our multigroup analyses found that awareness of CVI efforts in one’s neighborhood weakened the association between GVE and psychological distress.

This study adds to the growing body of research that has found a connection between GVE and adverse mental health symptoms. While some studies examine links between GVE and mental health using indices that capture social proximity to exposure [9], many focus on specific types of exposure, such as hearing gunshots, residing near a shooting incident, or non-fatal victimization. We found that more direct or socially proximal GVE was associated with increased psychological distress. This finding, while not providing causal evidence, aligns with Semenza and Kravitz-Wirtz’s tiered GVE framework [3], which posits that stress responses and well-being indicators are more likely to be impacted by more proximal gun violence exposures.

More direct GVE was also associated with a greater perceived need to carry a firearm for protection, which is consistent with other research on discrete or cumulative forms of GVE and gun carrying [17, 18, 20]. This link is illustrative of the potentially cyclical process by which gun violence triggers more gun violence (see Buggs et al. [45]), particularly within social networks [46, 47]. Among residents with greater GVE, perceptions of firearm carrying or other self-help strategies to ensure safety may be more common [48]. The potential for greater carriage and lethal retaliatory violence within communities already experiencing high rates of gun violence is reflected in one of the key goals of many CVI programs—to change norms around violence and gun use [31, 35].

The association between GVE and the perceived need to carry a firearm was partially mediated by anticipation of a future assault; however, the primarily direct effect of GVE (80% of the total effect) suggests that the association largely operates independent of specific assault anticipation. The extent to which firearm carrying is perceived as necessary may be more directly tied to past GVE and a broadly perceived need for protection rather than indirectly through anticipated assault risk. In other words, while there is a positive relationship between GVE and anticipated assault risk, and likewise a positive relationship between anticipated assault risk and the perceived need to carry a firearm, change in the perceived likelihood of being assaulted was not associated with a strong mediating effect.

A key contribution of this study is its examination of whether CVI awareness is associated with a weaker relationship between GVE and mental health and safety-related perceptions. The positive association between GVE and psychological distress was significantly weaker among those who reported awareness of local CVI efforts than those who were not aware of CVI. Although not statistically significant, similar differences existed for the relationships between GVE and firearm carrying perceptions, anticipated assault and firearm carrying perceptions, and anticipated assault and psychological distress. These findings suggest that the moderating role of CVI on mental well-being, safety perceptions, and firearm-carrying norms may extend to those not directly engaged by outreach workers but aware of CVI efforts.

While the precise mechanism through which CVI awareness may weaken the association between GVE and psychological distress is unknown, research on place-based interventions offers potential explanations. Although CVI does not involve changes to the physical environment, program activities may be associated with similar psychological benefits as vacant land remediation, if not also influence community-level social processes. Research has highlighted the benefits to mental health and perceptions of community associated with place-based interventions [36–38]. In the same manner that community-engaged neighborhood revitalization efforts signaled to residents that people “cared about their neighborhood” (Rupp et al. [37], p. 103), CVI may convey a similar signal. If program activities are perceived as active efforts to combat violence and improve community life, such a perception may attenuate the relationship between GVE and psychological distress.

CVI organizations may also play a larger, more active role in fostering community well-being through activities apart from violence interruption and mediation [31]. For example, outreach workers in Chicago provided economic and material aid to the broader community during the COVID-19 pandemic [49]. The impacts of other organized activities, such as peace marches, vigils, and firearm lockbox giveaways, are likewise not limited to CVI participants [34]. The relational nature of these public events illustrates the potentially diffuse impact that CVI may have, specifically through building collective efficacy in networks beyond (and including) those in which violence clusters. Indeed, community engagement and outreach efforts to support those not enrolled in programs have been identified as a key theory of change activity for CVI [34]. In Detroit, support for community-centered work to reduce violence is high; 96% of survey respondents indicate that it is somewhat or very important to have community-led programs working to reduce violence [50].

Our findings suggest that CVI may have indirect benefits beyond the violence-centric outcomes that are typically assessed. Given the potential moderating effect of CVI awareness on the relationship between GVE and psychological distress, efforts to increase community CVI awareness may be warranted. Research aimed at identifying the mechanisms through which CVI awareness moderates the relationship between GVE and adverse outcomes is needed to more fully characterize the potential benefits of these interventions. In particular, social network analyses could provide additional insight by beginning to characterize how the health consequences of GVE and CVI awareness flow through networks.

Although age was significantly associated with the perceived need to carry in a sensitivity analysis (Figure A2), its inclusion did not substantively change the magnitude or significance of primary path coefficients, suggesting that it did not strongly confound the relationships of interest. The substantially worse model fit in unweighted analyses reflects that a single direct path to perceived need to carry left covariances unmodeled, given that age was modestly correlated with all four key variables (*r* = −0.13 to −0.20). Although age is theoretically relevant to the relationships we explored, its exclusion was driven by worse model fit and considerations of model parsimony. Future research adopting a broader theoretical scope that fully specifies the roles of age and other factors across model variables would be valuable.

### Limitations

This study is not without limitations. First, the cross-sectional design limited causal inference, as we could not establish temporal ordering. Although the relationships we examined are consistent with conceptual [3] and theoretical [25] frameworks, the directionality of these relationships cannot be confirmed with cross-sectional data, and some relationships may be bi-directional. Second, our analysis may have been underpowered to detect group-level differences given the smaller sample and large standard errors associated with the CVI awareness group. Nonetheless, one relationship significantly differed between groups, and there were meaningful differences in the magnitudes of other coefficients, suggestive of moderation. Relatedly, although weighted least squares estimation would be preferred given non-normal distributions of two endogenous variables, this approach is unavailable with survey-weighted data in Stata. Sensitivity analyses using asymptotic distribution-free estimation on unweighted data produced nearly identical path coefficients and standard errors, suggesting distribution violations did not materially impact our estimates. Third, although the use of a representative citywide sample constitutes a strength of this study, we did not have access to geolocated data to determine neighborhood-level violence or residence in a CVI zone. However, CVI services extended beyond formal zones [39], suggesting that awareness of CVI activities could also exist across the city. To the extent that our awareness variable did not accurately reflect the presence of CVI in residents’ neighborhoods, our results may underestimate the true moderating role of CVI. Alternatively, if awareness instead reflected greater civic or community engagement, rather than awareness of CVI in one’s neighborhood, our results would overestimate the moderating role of CVI. Finally, as we focused on individual-level relationships in a single city, we were unable to examine how GVE contributes to broader community-level inequities [3]. Future research that incorporates data across municipalities can expand on our findings by investigating whether these relationships vary by macro-level factors.

## Conclusion

This study offers further support that more direct GVE is associated with worse mental well-being and negative safety-related perceptions, including the perceived need to carry a firearm for protection. At the same time, the results suggest that CVI may play a meaningful role in moderating the relationships between GVE and these outcomes, such as the association between GVE and psychological distress. In addition to existing outreach activity, strategies to increase CVI awareness may be warranted. Additional research that examines whether CVI influences the well-being and perceptions of those enrolled in CVI programs and those with no direct involvement is needed to more fully characterize the potential benefits of CVI.

## Supplementary Information

Below is the link to the electronic supplementary material.

Supplementary file1 (DOCX 388 kb)
