# Supplementary material for: Gun Violence Exposure and the Perceived Need to Carry a Firearm: The Role of Community Violence Intervention Awareness
Source: J Urban Health. 2026 Jul 31;103(5):1022–33. doi: 10.1007/s11524-026-01115-w (PMC13463175; doi:10.1007/s11524-026-01115-w)
Supplement: Supplementary file 1 [file 11524_2026_1115_MOESM1_ESM.docx]

**SUPPLEMENTAL MATERIAL**

**Table A1**. Direct and indirect path estimates in the overall and multigroup models using a three-group categorization.

|  | Group A:  No Violence Reduction Program Awareness | |  | Group B:  Violence Reduction Program Awareness | |  | Group C:  CVI Awareness | |
| --- | --- | --- | --- | --- | --- | --- | --- | --- |
|  | b | p |  | b | p |  | b | p |
| **Psychological distress** |  |  |  |  |  |  |  |  |
| GVE → Psychological distress | 0.22 | < .001 |  | 0.24 | .001 |  | 0.04 | .64 |
| Anticipated assault → Psych. distress | 0.14 | .001 |  | 0.18 | .001 |  | 0.04 | .49 |
| GVE → Anticipated assault → Psych. distress | 0.06 | .001 |  | 0.09 | .004 |  | 0.02 | .50 |
|  |  |  |  |  |  |  |  |  |
| **Anticipated assault risk** |  |  |  |  |  |  |  |  |
| GVE → Anticipated assault risk | 0.44 | < .001 |  | 0.52 | < .001 |  | 0.38 | < .001 |
|  |  |  |  |  |  |  |  |  |
| **Perceived need to carry** |  |  |  |  |  |  |  |  |
| GVE → Carry | 0.40 | < .001 |  | 0.60 | < .001 |  | 0.27 | .13 |
| Psychological distress → Carry | 0.07 | .20 |  | 0.12 | .22 |  | 0.03 | .81 |
| Anticipated assault risk → Carry | 0.18 | .001 |  | 0.16 | .06 |  | 0.05 | .66 |
| GVE → Psych. distress → Carry | 0.02 | .23 |  | 0.03 | .24 |  | 0.00 | .83 |
| GVE → Anticipated assault → Carry | 0.08 | .003 |  | 0.08 | .07 |  | 0.02 | .66 |
| GVE → Anticipated assault → Psych. distress → Carry | 0.00 | .23 |  | 0.01 | .26 |  | 0.00 | .81 |
| SRMR | 0.036 | | | | | | | |

*Note*. Goodness of fit statistics for the unweighted but identically specified model are as follows: RMSEA=0.063, CFI=0.937, TLI=0.843.

**Figure A1**. Multigroup structural equation model of the perceived need to carry a firearm among those who did not report awareness of CVI in their neighborhood (Group 1) and those who reported CVI awareness (Group 2) with additional paths from educational attainment.

Group 1: No CVI Awareness

Group 2: CVI Awareness

SRMR=0.016; Goodness of fit statistics for the unweighted but identically specified model are as follows: RMSEA=0.040, CFI=0.982, TLI=0.934

**Figure A2**. Multigroup structural equation model of the perceived need to carry a firearm among those who did not report awareness of CVI in their neighborhood (Group 1) and those who reported CVI awareness (Group 2) with age as a control.

Group 1: No CVI Awareness

Group 2: CVI Awareness

SRMR=0.044; Goodness of fit statistics for the unweighted but identically specified model are as follows: RMSEA=0.087, CFI=0.854, TLI=0.672

**Table A2**. Comparison of maximum likelihood and asymptotic distribution-free estimates from the overall model using unweighted data.

|  | Maximum Likelihood Estimation (MLE) | |  | Asymptotic Distribution-Free (ADF) | |  |
| --- | --- | --- | --- | --- | --- | --- |
|  | *b* | *se* |  | *b* | *se* |  |
| **Psychological distress** |  |  |  |  |  |  |
| GVE → Psychological distress | 0.17 | 0.03 |  | 0.18 | 0.03 |  |
| Anticipated assault → Psych. distress | 0.15 | 0.02 |  | 0.14 | 0.02 |  |
| GVE → Anticipated assault → Psych. distress | 0.07 | 0.01 |  | 0.06 | 0.01 |  |
|  |  |  |  |  |  |  |
| **Anticipated assault risk** |  |  |  |  |  |  |
| GVE → Anticipated assault risk | 0.44 | 0.03 |  | 0.44 | 0.03 |  |
|  |  |  |  |  |  |  |
| **Perceived need to carry** |  |  |  |  |  |  |
| GVE → Carry | 0.39 | 0.04 |  | 0.39 | 0.04 |  |
| Psychological distress → Carry | 0.10 | 0.03 |  | 0.11 | 0.03 |  |
| Anticipated assault risk → Carry | 0.24 | 0.03 |  | 0.24 | 0.03 |  |
| GVE → Psych. distress → Carry | 0.02 | 0.01 |  | 0.02 | 0.01 |  |
| GVE → Anticipated assault → Carry | 0.10 | 0.01 |  | 0.11 | 0.01 |  |
| GVE → Anticipated assault →  Psych. distress → Carry | 0.01 | 0.00 |  | 0.01 | 0.00 |  |
| SRMR | 0.033 | |  | 0.033 | |  |

*Note*. Goodness of fit statistics for unweighted models (as ADF estimation is unavailable with survey-weighted data in Stata): MLE – RMSEA=0.063, CFI=0.934, TLI=0.836; ADF – RMSEA=0.066, CFI=0.880, TLI=0.699.
